# Supplementary material for: The TRIM-NHL protein NHL-2 is a co-factor in the nuclear and somatic RNAi pathways in C. elegans
Source: eLife. 2018 Dec 21;7:e35478. doi: 10.7554/eLife.35478 (PMC6351104; doi:10.7554/eLife.35478)
Supplement: Transparent reporting form [file elife-35478-transrepform.pdf]

eLIFE
1st Floor
24 Hills Road
Cambridge CB2 1JP, UK
P 01223 855340
W elifesciences.org
T @elife

eLife's transparent reporting form

We encourage authors to provide detailed information *within their submission* to facilitate the interpretation and replication of experiments. Authors can upload supporting documentation to indicate the use of appropriate reporting guidelines for health-related research (see EQUATOR Network), life science research (see the BioSharing Information Resource), or the ARRIVE guidelines for reporting work involving animal research. Where applicable, authors should refer to any relevant reporting standards documents in this form.

If you have any questions, please consult our Journal Policies and/or contact us: editorial@elifesciences.org.

Sample-size estimation

- You should state whether an appropriate sample size was computed when the study was being designed
- You should state the statistical method of sample size computation and any required assumptions
- If no explicit power analysis was used, you should describe how you decided what sample (replicate) size (number) to use

Please outline where this information can be found within the submission (e.g., sections or figure legends), or explain why this information doesn't apply to your submission:

Accompanying the description of the experimental procedures in the EXPERIMENTAL PROCEDURES section we have referenced articles on which the assays in figures 1-3 were based on. Sample size was determined by the accepted practice in the field.

Replicates

- You should report how often each experiment was performed
- You should include a definition of biological versus technical replication
- The data obtained should be provided and sufficient information should be provided to indicate the number of independent biological and/or technical replicates
- If you encountered any outliers, you should describe how these were handled
- Criteria for exclusion/inclusion of data should be clearly stated
- High-throughput sequence data should be uploaded before submission, with a private link for reviewers provided (these are available from both GEO and ArrayExpress)

Please outline where this information can be found within the submission (e.g., sections or figure legends), or explain why this information doesn't apply to your submission:

Experiments in figures 1, 2, 3, had at least two biological replicates, as described in the text/figure legends. We defined biological replicates as experiments in which the worms were grown and tested independently of each other.

Illumina sequencing libraries (small RNA, mRNA-seq):

- Triplicate biological replicates were sequenced at each temperature studied, as noted in the Experimental Procedures Section.

Computational Analysis of seq data:

- No outliers
- In Section "ACCESION NUMBERS": All small RNA and mRNA Illumina sequencing data have been submitted to the NCBI's Sequence Read Archive (SRA), and are included under project accession number SRP115391

Statistical reporting

- Statistical analysis methods should be described and justified
- Raw data should be presented in figures whenever informative to do so (typically when N per group is less than 10)
- For each experiment, you should identify the statistical tests used, exact values of N, definitions of center, methods of multiple test correction, and dispersion and precision measures (e.g., mean, median, SD, SEM, confidence intervals; and, for the major substantive results, a measure of effect size (e.g., Pearson's r, Cohen's d)
- Report exact p-values wherever possible alongside the summary statistics and 95% confidence intervals. These should be reported for all key questions and not only when the p-value is less than 0.05.

Please outline where this information can be found within the submission (e.g., sections or figure legends), or explain why this information doesn't apply to your submission:

- Brood size, somatic RNAi and germline mortality data (Figures. 1-3) where graphed as mean and standard deviation, and statistical significance examined use ANOVA tests. Prism7 software was used as indicated in the EXPERIMENTAL PROCEDURES.
- The statistical test for gene set overlap was done by hypergeometric test with p-values corrected by a FDR procedure (divided by the ranks of each p-value and multiplied by the number of multiple tests, in Figure 5,7, as described in "EXPERIMENTAL PROCEDURES" – "Small RNA Cloning and Data Analysis".
- The exact corrected p-value (or indicated as q-value) for each test mentioned in the text (wherever Figure 5,7 was referred to) was given accordingly. Due to numeric calculation precision limits, very small p-values were indicated as q < 1E-300.

(For large datasets, or papers with a very large number of statistical tests, you may upload a single table file with tests, Ns, etc., with reference to sections in the manuscript.)

Group allocation

- Indicate how samples were allocated into experimental groups (in the case of clinical studies, please specify allocation to treatment method); if randomization was used, please also state if restricted randomization was applied
- Indicate if masking was used during group allocation, data collection and/or data analysis

Please outline where this information can be found within the submission (e.g., sections or figure legends), or explain why this information doesn't apply to your submission:

C. elegans samples were split into wild-type or mutant groups, as detailed throughout the manuscript, along with the temperature they are grown at (Experimental Procedures, main text, and figure legends.).

Additional data files ("source data")

- We encourage you to upload relevant additional data files, such as numerical data that are represented as a graph in a figure, or as a summary table
- Where provided, these should be in the most useful format, and they can be uploaded as "Source data" files linked to a main figure or table
- Include model definition files including the full list of parameters used
- Include code used for data analysis (e.g., R, MatLab)
- Avoid stating that data files are "available upon request"

eLife Sciences Publications, Ltd is a limited liability non-profit non-stock corporation incorporated in the State of Delaware, USA, with company number 5030732, and is registered in the UK with company number FC030576 and branch number BR015634 at the address 1st Floor, 24 Hills Road, Cambridge CB2 1JP | August 2014

eLIFE
1st Floor
24 Hills Road
Cambridge CB2 1JP, UK
P 01223 855340
W elifesciences.org
T @elife

Please indicate the figures or tables for which source data files have been provided:

- The R code used for the venn-pie diagrams (Figure 5,7) is attached.

eLife Sciences Publications, Ltd is a limited liability non-profit non-stock corporation incorporated in the State of Delaware, USA, with company number 5030732, and is registered in the UK with company number FC030576 and branch number BR015634 at the address 1st Floor, 24 Hills Road, Cambridge CB2 1JP | August 2014
